# Role of leaf volatiles in spotted-wing drosophila (Diptera: Drosophilidae) attraction to blueberry fruits

**DOI:** 10.1093/jisesa/ieag053

**Published:** 2026-06-16

**Authors:** Kevin R Cloonan, Pablo Urbaneja-Bernat, Caitlin C Rering, Alexia M Lanier, Beth Ferguson, Yahel Ben-Zvi, Cesar Rodriguez-Saona

**Affiliations:** USDA-ARS, Subtropical Horticulture Research Station, Miami, FL, USA; Trécé Incorporated, Adair, OK, USA; Plant Immunity and Biochemistry Group, Department of Biology Biochemistry and Natural Sciences, Universitat Jaume I, Castelló de la Plana, Spain; USDA-ARS, Chemistry Research Unit, Center for Medical, Agricultural, and Veterinary Entomology, Gainesville, FL, USA; USDA-ARS, Chemistry Research Unit, Center for Medical, Agricultural, and Veterinary Entomology, Gainesville, FL, USA; Philip E. Marucci Center for Blueberry & Cranberry Research, Rutgers University, Chatsworth, NJ, USA; Philip E. Marucci Center for Blueberry & Cranberry Research, Rutgers University, Chatsworth, NJ, USA; Philip E. Marucci Center for Blueberry & Cranberry Research, Rutgers University, Chatsworth, NJ, USA

**Keywords:** *Drosophila suzukii*, behavior, semiochemical, volatile organic compound, monitoring, integrated pest management

## Abstract

Herbivorous insects use volatile organic compounds (VOCs) to find food, mates, and oviposition sites. Understanding these cues is key for behavior-based pest management. Spotted-wing drosophila (*Drosophila suzukii* Matsumura) (Diptera: Drosophilidae) is an invasive pest that attacks ripening fruit. Although fruit VOCs attract this species, the role of nonfruit plant volatiles, such as those emitted by leaves, remains unclear. Here, laboratory and semifield behavioral assays were used to test whether leaf volatiles enhance adult *D. suzukii* attraction and oviposition on blueberry fruit, followed by gas chromatography-mass spectrometry characterization of fruit and leaf VOCs and behavioral and electroantennographic (EAG) assays to assess responses to individual compounds. In laboratory trials, both sexes were significantly more attracted to fruit when leaves were present compared to fruit alone. Likewise, oviposition rates increased in the presence of leaves during cage choice experiments. Leaves were more fragrant than fruit on a per gram basis, emitting both a greater quantity and number of compounds. Among the VOCs emitted in greater amounts from the “fruit + leaf” treatment than from “fruit” alone, 9 compounds were examined, and 2—linalool and 6,10,14-trimethyl-2-pentadecanone—attracted females individually and in a blend and elicited strong EAG responses. These findings demonstrate that attraction is influenced by a combination of plant-derived cues; however, further field studies are needed to confirm these results under natural conditions. This knowledge advances our understanding of *D. suzukii* chemical ecology and may support the development of improved monitoring tools and behavior-based control strategies for this pest.

## Introduction

Habitat selection by herbivorous insects is a critical first step in locating suitable hosts for feeding, mating, and oviposition, which ultimately ensures their survival and reproductive success (Carrasco et al. 2015). Host-plant volatile organic compounds (VOCs) play a central role in this process, as herbivorous insects rely on specific chemical cues to detect and navigate toward preferred habitats and hosts (Bruce et al. 2005, Szendrei and Rodriguez-Saona 2010, Bruce and Pickett 2011, Webster and Cardé 2017). Insights into these VOCs offer opportunities to manipulate insect behavior for sustainable integrated pest management (IPM) (Shrivastava et al. 2010, Szendrei and Rodriguez-Saona 2010).

Spotted-wing drosophila, *Drosophila suzukii* Matsumura (Diptera: Drosophilidae), is an invasive pest that attacks soft, thin-skinned fruits, including blueberry, raspberry, strawberry, and cherry (Lee et al. 2011, Walsh et al. 2011, Asplen et al. 2015, Tait et al. 2021, Nair and Peterson 2023). Native to Southeast Asia, *D. suzukii* has rapidly expanded its range over the past 2 decades, establishing in North America, Europe, and parts of South America and Oceania (Kirschbaum et al. 2021). Unlike most *Drosophila* species that lay eggs on overripe or decaying fruit, *D. suzukii* females possess a uniquely enlarged, serrated ovipositor that allows them to lay eggs inside ripening, marketable fruit (Atallah et al. 2014). This behavior results in early fruit damage and significant crop losses, making *D. suzukii* especially challenging to manage using conventional pest control strategies (Lee et al. 2011).

Although monitoring tools for *D. suzukii* are available (Lee et al. 2011, Landolt et al. 2012, Cha et al. 2018, Cloonan et al. 2018), most lures are based on fermentation-derived VOCs, such as acetic acid, ethanol, acetoin, ethyl octanoate, and methionol (Landolt et al. 2012, Cha et al. 2013, 2014, 2015, 2017, 2018, Kleiber et al. 2014, Akasaka et al. 2017, Jaffe et al. 2018, Larson et al. 2021). A major limitation of these attractants is their low specificity, often resulting in high bycatch of nontarget insects (Basoalto et al. 2013, Burrack et al. 2015, Mazzetto et al. 2015, Hamby and Becher 2016, Tonina et al. 2018). For instance, commercial lures containing these compounds typically exhibit only 15%–60% specificity for *D. suzukii* (Cloonan et al. 2019, Larson et al. 2021). Therefore, identifying host-associated VOCs involved in habitat selection and host location could improve both the efficacy and selectivity of monitoring tools.

Beyond monitoring, these VOCs may also support the development of behavior-based management strategies. Currently, *D. suzukii* control relies largely on calendar-based insecticide applications (Haviland and Beers 2012, Diepenbrock et al. 2016, Farnsworth et al. 2017, Iglesias and Liburd, 2017, Hunter and Sial, 2019, Joshi et al. 2023). Although IPM tactics such as attract-and-kill and other behavior-modifying approaches have been explored (Haye et al. 2016, Ibouh et al. 2019, Tait et al. 2021, Spitaler et al. 2022, Nair and Peterson 2023, Duménil et al. 2025a), their practical implementation and widespread adoption remain limited, likely due to the absence of highly specific attractants. Advancing these behavior-based strategies will require a deeper understanding of *D. suzukii’*s sensory ecology, particularly the role of VOCs in host-location behavior.

Like many herbivorous insects (Bruce and Pickett 2011, Webster and Cardé 2017), *D. suzukii* relies heavily on olfactory cues to locate hosts and select oviposition sites (Keesey et al. 2015, Revadi et al. 2015, Cloonan et al. 2018). Because *D. suzukii* moves continuously between noncrop habitats and crops throughout the season (Urbaneja-Bernat et al. 2020, Buck et al. 2022), identifying host-plant cues is critical for monitoring and management. The species responds to VOCs emitted not only from fruit (Abraham et al. 2015, Cai et al. 2019) but also from leaves (Keesey et al. 2015, Piñero et al. 2019) and associated microbes (Hamby et al. 2012, Bueno et al. 2020, Jones et al. 2021). For example, leaf-derived VOCs such as β-cyclocitral may facilitate habitat location before females engage in more refined host-searching behavior guided by fruit and microbial volatiles. β-cyclocitral is an important leaf-derived volatile mediating *D. suzukii* attraction in strawberries (Keesey et al. 2015). Although responses to individual odor sources have been studied, little is known about how *D. suzukii* integrates cues from multiple odor sources during habitat and host selection (Keesey et al. 2015, Cloonan et al. 2019, Duménil et al. 2025a). Because oviposition occurs in fruit, it is plausible that leaf-emitted VOCs enhance female attraction to fruit, yet this hypothesis remains largely untested. Notably, female *D. suzukii* exhibited significantly greater attraction to fruit juice supplemented with β-cyclocitral than to fruit juice alone (Piñero et al. 2019).

This study examines the combined effects of fruit and leaf volatiles from highbush blueberry (*Vaccinium corymbosum* L.) on *D. suzukii* attraction and oviposition. We hypothesize that the presence of leaf volatiles enhances *D. suzukii* attraction to fruit. Using laboratory, greenhouse, and semifield experiments supported by chemical analyses, we aimed to: (i) test whether the presence of leaves increases attraction and oviposition in fruit; (ii) identify VOCs emitted by blueberry fruit and leaves; and (iii) evaluate selected VOCs for their behavioral and electrophysiological (electroantennogram, EAG) effects on *D. suzukii*. Although field validation is required before implications for monitoring or IPM strategies can be drawn, these findings contribute to our understanding of how leaf-derived volatiles may influence *D. suzukii* attraction to blueberry fruit under laboratory and semifield conditions.

## Materials and Methods

### Insects

*Drosophila suzukii* used in this study originated from a laboratory colony established in 2013 using wild flies collected in Atlantic County, New Jersey (United States). The colony has been maintained at the Rutgers P.E. Marucci Center (Chatsworth, New Jersey) on a standard artificial diet following the protocols of Dalton et al. (2011) and Jaramillo et al. (2015). Flies were reared in 50-ml polystyrene vials (Fisher Scientific, Nazareth, Pennsylvania, United States) containing ∼15 ml of diet and sealed with BuzzPlugs (Fisher Scientific). The colony was kept in an incubator (Percival Scientific, Perry, Iowa, United States) under controlled conditions (25 °C, 55% relative humidity, and a 16:8 h light:dark photoperiod). To maintain genetic diversity, field-collected individuals were introduced annually into the colony. For the assays, 3 to 7-d-old male and female flies were used to ensure sexual maturity (Tochen et al. 2014). Prior to testing in olfactory assays, flies were food-deprived for ∼8 h to enhance responsiveness to VOCs. During the food deprivation period, flies had access to water via a 3.8-cm dental wick soaked in deionized water.

### Plant Tissue Sampling and Handling

Plant material from highbush blueberries (*V. corymbosum* var. “Bluecrop”) used for the studies and volatile collections was obtained from mature 3 to 4-year-old bushes planted in 2018 to 2019 at the Rutgers P.E. Marucci Center in Chatsworth, New Jersey (United States), where the laboratory and semifield bioassays were also conducted. Terminals containing fruit clusters alone, leaves alone, or fruits with leaves were prepared and bagged at the green-fruit stage using gauze bags (18 × 42 × 48 cm; Temkin International, Springville, Utah, United States) to prevent natural infestation, with no insecticides applied. Branches were inspected for damage prior to bagging, and only healthy branches were selected. For laboratory bioassays, fruits were hand-picked, and leaves were excised at the base of the petiole using surgical scissors. In the semifield oviposition study, fruit clusters and leaves were excised from the peduncle and placed in floral water picks to prevent desiccation. All plant tissues were used on the day of collection and transported in a cooler. Volatile collections in the field were conducted in situ, without removing fruit or leaves from the plants.

### Chemicals

The VOCs (*Z*)-3-hexenyl acetate (≥98%, CAS No. 3681-71-8), linalool (97%, CAS No. 78-70-6), methyl benzoate (>99%, CAS No. 93-58-3), farnesene (mixture of isomers, CAS No. 502-61-4), ocimene (mixture of isomers, CAS No. 13877-91-3), 6-methyl-5-heptene-2-one (≥98%, CAS No. 110-93-0), citral (95%, CAS No. 5392-40-5), and acetoin (≥96%, CAS No. 513-86-0) were purchased from Sigma-Aldrich (St. Louis, Missouri, United States). Methyl salicylate (>99%, CAS No. 119-36-8) was purchased from Fluka Chemie GmbH (Buchs, Switzerland), and 6,10,14-trimethyl-2-pentadecanone (≥98%, CAS No. 502-69-2) was purchased from Synthonix Inc. (Wake Forest, North Carolina, United States). The solvent dichloromethane (≥99.8%, CAS No. 75-09-2) was purchased from Sigma-Aldrich.

### Olfactory Experiments with Fruit and Leaf Material

**Fig. 1.**
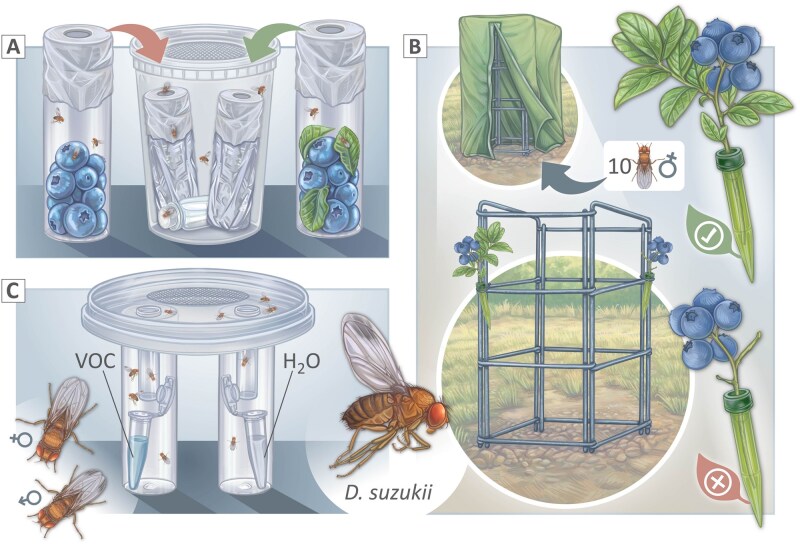
Setup of behavioral assays. A) Choice olfactory assays conducted in the laboratory. B) Semifield oviposition assays. C) Dose–response assays conducted in the greenhouse. Artist credit: Lindsay Lindhult, made using Procreate and Adobe Illustrator.

Experiments were conducted to assess the olfactory response of *D. suzukii* to blueberry fruit in the presence and absence of blueberry leaves. We used a dual-choice assay following the general methodology described by Urbaneja-Bernat et al. (2021). The experimental arena consisted of a transparent polypropylene cylindrical plastic cup (946 ml; 114 mm diameter × 127 mm height; Paper Mart, California, United States) with a circular hole (8 cm diameter) cut into the lid and covered with nylon mesh for ventilation (Fig. 1A). This arena size has been shown to be sufficient for studying *D. suzukii* attraction to volatiles in previous work (Feng et al. 2018, Urbaneja-Bernat et al. 2021, Rering et al. 2023). Two 50-ml polystyrene vials (95 mm height × 28.5 mm diameter), identical to those used in insect rearing (see above) and containing the treatment choices, were positioned vertically on opposite sides of the cup.

We conducted 4 choice tests: (i) “blank” vs “fruit,” (ii) “blank” vs “leaf,” (iii) “blank” vs “fruit + leaf,” and (iv) “fruit” vs “fruit + leaf.” The “leaf” treatment consisted of 4 to 5 fully developed blueberry leaves (∼0.5 g); the “fruit” treatment contained ∼5 g of ripe (blue) blueberries; the “fruit + leaf” treatment included both; and the “blank” treatment contained neither leaves nor fruit. Plant material was collected in June–July as described above. To eliminate visual cues, all polystyrene vials were wrapped in aluminum foil. Vial openings were sealed with Parafilm (Amcor, Zürich, Switzerland), leaving a 4-mm central hole to allow fly entry but restricting their exit. To maintain humidity within the arena, a 20-ml glass vial filled with deionized water and plugged with a cotton ball was placed at the center of each setup.

Ten adult *D. suzukii* (5 males and 5 females) were released into the center of each arena, and their positions were recorded after 24 h to assess preference. The number of flies in each vial was used as a measure of attraction to the respective treatment. Assays were conducted inside a fume hood under controlled conditions: 25 ± 1 °C, 60 ± 5% relative humidity (RH), a 16:8 h light:dark photoperiod, and ∼1700 lux light intensity. There were 10 replicates per choice combination. The experiment was conducted 5 times with new flies, and 8 randomly selected choice combinations were evaluated concurrently at each time point. The position of the vials (left or right) was alternated systematically to avoid positional bias.

### Oviposition Experiments with Fruit and Leaf Material

To evaluate the effect of leaf presence on *D. suzukii* oviposition in blueberry fruit, we conducted an experiment in insect-proof outdoor mesh enclosures (1.83 × 1.83 × 1.83 m; Outdoor Cage Mesh Enclosures, BioQuip Products, Rancho Dominguez, California, United States). Cages were randomly placed in an open field bordered by woods (25 ± 2 °C, 75 ± 5% RH), with at least 10 m between them. Each cage contained 2 blueberry fruit clusters—either both of the same treatment (no-choice tests) or 1 with leaves and 1 without (choice tests)—suspended from galvanized steel wire square tomato cage at equal height (∼1 m) and distance (∼50 cm) (Fig. 1B). Ripe fruit clusters (each containing ∼10 fruits, ∼20 to 25 g) were collected in June–July 2019 from blueberry bushes that had been bagged at the green-fruit stage (as described above). In the “fruit + leaf” treatment, clusters included 8 to 10 attached leaves (∼1.1 g) and were randomly assigned to positions within each cage; the “fruit” treatment consisted of clusters without leaves. Ten sexually mature *D. suzukii* females were introduced into each cage between 17:00 and 18:00 h, having been removed from the colony 2 to 3 h prior to release. These hours were chosen because they occur after the peak daytime heat, reducing *D. suzukii* mortality, and providing a 24‑h exposure period without disrupting their natural diel activity patterns. After the 24-h exposure period, fruit clusters were collected, and individual berries were examined under a stereomicroscope to count eggs per berry. The choice experiment was repeated at 4 different time points using new flies, for a total of 10 replicates per no-choice test (“fruit” only and “fruit + leaf” only) and choice test (“fruit” vs “fruit + leaf”).

### Volatile Collection and Analysis

Headspace volatiles from the “fruit,” “leaf,” and “fruit + leaf” treatments were collected in situ using a pull system in June 2023 (*n* =5 replicates per treatment). Single branches of blueberry bushes (see “Plant tissue sampling and handling” section above) containing either ∼7 fruits (∼16.5 g; fully mature, blue color), ∼38 leaves (∼4.5 g), or both were enclosed in 20 × 20 cm volatile collection bags made of nonabsorbent Vac-Pak material (Richmond Products, Norwalk, California, United States). Branches were inspected prior to collection, and only healthy, undamaged branches were selected. Different bushes were used for each treatment and replicate. The bag openings around the stems were sealed with binder clips, and headspace volatiles were trapped on adsorbent filters by pulling air at 1 l min^−1^ with a 12 V vacuum pump (Sensidyne, St. Petersburg, Florida, United States). Filters consisted of glass tubes (3.5 cm length; 3/32″ i.d., 5/32″ o.d., 1/32″ wall) packed with ∼40 mg HayeSep Q Porous Polymer Adsorbent (80 to 100 mesh; Supelco, Bellafonte, Pennsylvania, United States), secured with 2 discs of fine metal mesh. Before use, filters were rinsed with ≥ 600 µl dichloromethane, dried under purified N_2_ for ∼2 min, and then wrapped in Kimwipes for padding and stored in individual amber glass vials. Collections were conducted for 24 h. Empty bags were sampled as controls (*n *=3) to monitor contamination. After collection, all plant material was harvested and weighed for fresh mass. Bags were rinsed with tap water followed by 70% ethanol before reuse.

Postsampling, filters were shipped overnight on dry ice to the USDA-ARS Center for Medical, Agricultural and Veterinary Entomology in Gainesville (Florida, United States) and immediately stored at −20 °C until extraction and analysis. One leaf sample was lost during elution. Filters were eluted with dichloromethane containing 10 ng µl cis-jasmone as a surrogate standard to assess extraction consistency. Volatiles were eluted by gravity into autosampler vials with 250 µl inserts using 300 µl of the elution solution, followed by a brief high-purity N_2_ purge to recover any remaining eluent. Preliminary tests confirmed complete solubilization of trapped volatiles. Volatile extracts were analyzed immediately using an Agilent, 7890B; gas chromatograph coupled to a 5977; mass spectrometer (GC-MS; Agilent Technologies, Santa Clara, California, United States). Helium was the carrier gas. A 1 µl aliquot was injected via autosampler into an inlet held at 230 °C in splitless mode (56 ml min^−1^) with a purge at 0.75 min (30 ml min^−1^). Separation was achieved on a DB-Wax column (60 m × 320 µm × 0.25 µm; Agilent) at a constant flow of 3 ml min^−1^. Initially, the oven temperature was 30 °C and was ramped at 4 °C min^−1^ to a final temperature of 190 °C over 40 min, and then held at 230 °C for 2 min postrun. The electron ionization source operated at 70 eV, the MS transfer line and source at 230 °C, and the quadrupole at 150 °C. MS data were acquired in scan mode (40 to 400*m/z*, 2.1 scans s^−1^) with a 5.5 min solvent delay.

Chromatograms were manually analyzed to distinguish sample-derived volatiles from background contaminants. Features unique to samples were quantified using MassHunter Quantitative Analysis (Agilent). Quantitative and qualifying ions are listed in Supplementary Table S1. Volatiles were tentatively identified by matching fragmentation patterns to the NIST14 spectral database and comparing experimentally derived and PubChem retention indices. Two compounds could not be matched and are reported as unknown (Supplementary Table S1). The 9 VOCs selected for behavioral and electrophysiological (EAG) assays with *D. suzukii* (Table 1) were confirmed via coinjection with commercial standards and quantified via external calibration. These compounds were emitted in significantly greater amounts from the “fruit + leaf” treatment than from “fruit” alone (see “Results” section), are common in many plants attacked by *D. suzukii*, and may play a role in the pest’s chemical ecology (Abraham et al. 2015, Cloonan et al. 2018, Gale et al. 2024).

**Table 1.** Concentrations of volatile compounds that were significantly higher in the “fruit + leaf” treatment than in the “fruit” treatment (based on Supplementary Table S1) and selected for further testing

Compound	R.I. (polar)	Concentration (ng g^−1^; Mean ± SE)		
		Fruit	Leaf	Fruit + Leaf
**(*E*)-β-Ocimene**	1249	0	12.1 ± 7.1	6.0 ± 2.7
**(*Z*)-3-Hexenyl acetate**	1314	0.04 ± 0.04^a^	0.66 ± 0.43	2.5 ± 2.3
**6-Methyl-5-hepten-2-one**	1333	0.09 ± 0.09^a^	6.4 ± 3.5	4.2 ± 1.8
**Linalool**	1546	0.06 ± 0.06^a^	2.5 ± 0.8	1.9 ± 0.9
**Methyl benzoate**	1615	0.21 ± 0.04	5.1 ± 2.8	2.4 ± 1.7
**Citral**	1726	0	1.3 ± 0.8	0.47 ± 0.29
**(*E, E*)-α-farnesene**	1746	0	66 ± 37	49.4 ± 26.5
**Methyl salicylate**	1767	3.03 ± 0.22	108 ± 53	49.3 ± 21.3
**6,10,14-Trimethyl-2-pentadecanone**	2123	1.87 ± 0.32	24.0 ± 7.4	4.9 ± 2.3

a Compound detected in only 1 replicate.

### Olfactory Experiments with Synthetic VOCs

Based on VOC profiles collected from blueberry fruit and leaf material, we conducted greenhouse olfactory experiments to evaluate the attraction of *D. suzukii* to nine selected VOCs (Table 1). Because VOCs are highly volatile and their natural emission levels vary, we tested the compounds at a range of doses in our greenhouse experiments. Choice arenas (Fig. 1C) were constructed following the designs of Prokopy et al. (1995) and Rering et al. (2023), with minor modifications. Each arena consisted of a Petri dish (90 mm diameter × 15 mm height) with two 10-mm diameter holes drilled into the bottom plate, positioned 40 mm apart. Each hole was fitted with a small entrance straw that allowed flies to access a 50 ml polystyrene vial, identical to those used for insect rearing, placed beneath but prevented their escape. Each vial contained a 2-ml microcentrifuge tube (Eppendorf North America, Inc., Holstein, Germany) holding a piece of cotton roll (Dynarex Corporation, Orangeburg, New York, United States) impregnated with either a test VOC at one of 5 doses (2.5, 5.0, 10.0, 25.0, or 50.0 µl) of the neat compound or a blank control (without VOC). The 9 VOCs tested were (*Z*)-3-hexenyl acetate, ocimene, linalool, 6-methyl-5-hepten-2-one, citral, 6,10,14-trimethyl-2-pentadecanone, methyl salicylate, methyl benzoate, and farnesene. For each arena, treatment and control vials of the same dose were placed randomly on the left or right to avoid positional bias. Experiments were conducted in a greenhouse, at 22 ± 1 °C and 60 ± 5% RH under uniform overhead lighting for 5 h, to ensure adequate ventilation and prevent potential interference in cross-choice tests. Olfactory tests were conducted weekly over 2 months (from September to November 2025) with each volatile tested individually to avoid cross-contamination. Ten female *D. suzukii* were anesthetized on ice before being transferred to the arena, and the Petri dish was sealed with Parafilm to prevent escape. Females were used because they are responsible for fruit damage through oviposition; therefore, targeting female behavior is essential for preventing crop loss. Environmental conditions and fly survival were monitored at 1, 3, and 5 h to ensure consistency and minimize mortality. At the end of each trial, the number of flies captured in each vial was recorded. Each VOC–dose combination was replicated 8 times.

Based on the identification of the leaf volatiles linalool and 6,10,14-trimethyl-2-pentadecanone as attractants (see “Results” section), we conducted dual-choice assays to test linalool, 6,10,14-trimethyl-2-pentadecanone, and a blend of the 2 compounds (9.9:1 6,10,14-trimethyl-2-pentadecanone to linalool ratio, based on GC-MS data; see “Results” section) against an untreated (blank) control. The assays followed the design described in “Olfactory experiments with fruit and leaf material” section (Fig. 1A), except that 1 vial contained a microcentrifuge tube with 10 µl of either linalool, 6,10,14-trimethyl-2-pentadecanone, or the linalool–6,10,14-trimethyl-2-pentadecanone mixture, while the other vial contained a control (no VOC). The selected dose was chosen because it elicited behavioral responses in the greenhouse bioassays described above. Laboratory bioassays were run under direct overhead lighting set at 22 ± 1 °C, 60 ± 5% RH, and 16:8 h light:dark. Ten adult *D. suzukii* (5 males and 5 females) were released into the center of each arena, and their positions were recorded after 24 h to assess preference. The experiment was conducted as a single run with each treatment combination replicated 10 times.

### Electroantennographic Assays

In addition to behavioral assays, EAG experiments were conducted to compare female *D. suzukii* antennal responses to the nine selected VOCs (Table 1) with those to acetoin, a known attractant and antennally active compound (Cha et al. 2014, Cloonan et al. 2019). Stimulus cartridge preparation, antennal mounting, and EAG apparatus followed Cloonan et al. (2019). Stimulus applicators consisted of 14.5-cm glass Pasteur pipettes containing a 6 × 0.5-cm strip of filter paper loaded with 10 µl of each neat VOC, a dose shown to elicit behavioral responses for some, but not all, of the VOCs tested (see “Results” section). Acetoin was used as a standard reference VOC (positive control) to confirm that the antennae were properly prepared and responsive, while air served as the negative control. Recording and base electrodes were prepared by inserting silver wires into drawn capillary tubes filled with phosphate-buffered saline (NaCl, 4 g; Na_2_HPO_4_, 0.57 g; KH_2_PO_4_, 0.1 g; KCl, 0.1 g in 500 ml distilled water). To attach the base electrode, the abdomen, wings, and legs of each fly was removed and the saline-filled capillary tube inserted into the thoracic cavity. The recording electrode was then advanced toward the antenna with a micromanipulator until the antenna contacted the saline droplet. Preparations were continuously supplied with charcoal-filtered, humidified air at 1.5 l min^−1^. EAG signals were recorded using an IDAC-02 interface board and Syntech software (Syntech Ltd., Hilversum, The Netherlands). Stimuli were delivered at 10-s intervals with a 0.5-s pulse and 1-min spacing between VOCs. Responses were quantified as maximum depolarization amplitudes (mV), and values were normalized by subtracting the responses to the air (negative) controls. Each compound was replicated 10 times.

### Statistical Analysis

Data from behavioral studies were analyzed using SPSS Statistics v23.0 (SPSS Inc., Chicago, Illinois, United States) and JMP v14.2.0 (SAS Institute Inc., Cary, North Carolina, United States). Choice data from laboratory, greenhouse, and semifield (cage) experiments were analyzed using paired *t*-tests. No-choice data were analyzed with independent-samples *t*-tests. For each replicate, the mean response (proportion of *D. suzukii* in choice assays) or number of eggs per fruit was calculated before analysis. Normality and homogeneity of variances were assessed using the Shapiro–Wilk and Levene’s tests, respectively. Proportion data were arcsine square-root transformed prior to analysis, though untransformed values are shown in the figures. Differences among VOC doses were tested but were not significant for any compound and are therefore not reported. Data from EAG studies were analyzed using analysis of variance (ANOVA). Statistical significance was defined as *P *≤ 0.05.

Volatile data were analyzed in R (Version 4.4.1; R Core Team 2024) with RStudio (2024.09.0 + 375; Posit Team 2024). Peak areas were normalized by leaf and/or berry fresh weight, then log_10_(*x* + 1) transformed. Total volatile emission (sum of all normalized and transformed volatile peak areas) was compared between sample types with an ANOVA (function *aov* in the stats package). Pairwise comparisons between sample types were conducted using the TukeyHSD function in the stats package. Differences between samples were investigated using principal coordinate analysis (PCoA) with Bray–Curtis dissimilarities in package phyloseq (McMurdie and Holmes 2013) and with permutational multivariate analysis of variance (PERMANOVA) with the function *adonis2* in package vegan (Oksanen et al. 2024). Homogeneity of variance between sample group spatial medians was evaluated using the *betadisper* function in vegan. To identify which volatiles were more abundant in “fruit + leaf” mix samples than “fruit”-only samples, a differential analysis based on the negative binomial distribution was conducted with the function *DESeq* in the package DESeq2 (Love et al. 2014). For only the differential analysis, nonlog transformed (but fresh weight-normalized) peak areas were used, as this analysis is designed for large integer data, that is, read counts generated in high-throughput sequencing. Volatile data plots were prepared using ggplot2 (Wickham 2016).

## Results

### Olfactory Experiments with Fruit and Leaf Material

**Fig. 2.**
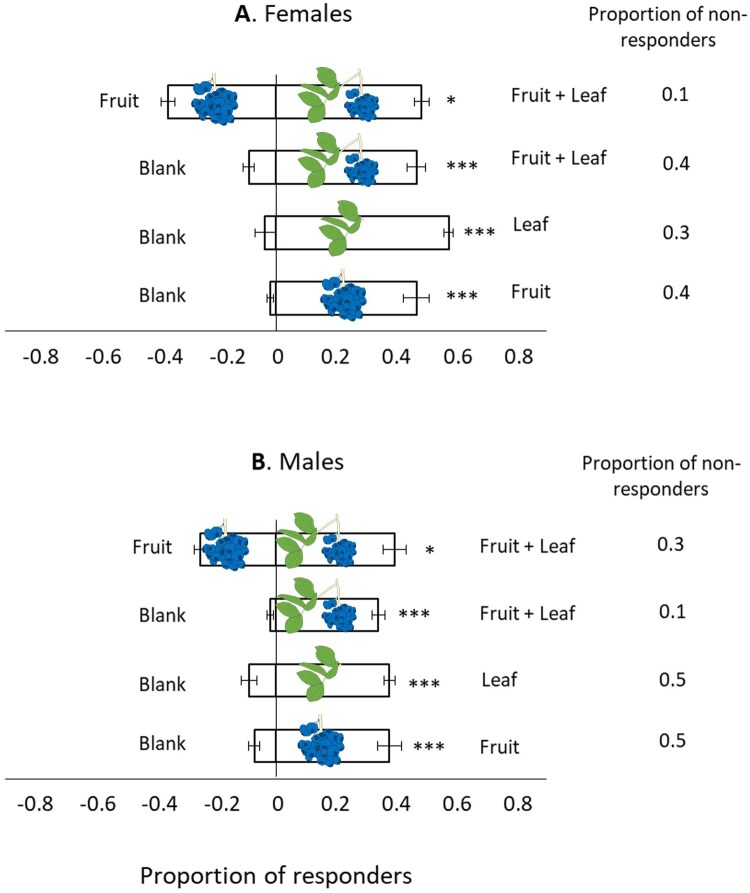
Mean (± SE) proportion of *Drosophila suzukii* females A) and males B) that made a choice between: fruit vs blank control, leaf vs blank control, fruit + leaf vs blank control, and fruit vs fruit + leaf in laboratory assays. Ten *D. suzukii* adults (5 males and 5 females) were released into the center of each arena (Fig. 1A). Significant differences between treatments (*t*-tests) are indicated as follows: ***P < 0.001 and *P < 0.05. *n *= 10.

In olfactory choice experiments, both female and male *D. suzukii* showed significant preferences for blueberry clusters with leaves compared to fruit alone or blank controls (Fig. 2). For females (Fig. 2A), the “fruit + leaf” combination elicited a significantly stronger response than “fruit” alone (*t *= 2.33; *df* = 9; *P *= 0.031). When tested against a blank, females also showed clear preferences for both “fruit + leaf” (*t *= 6.68; *df* = 9; *P *< 0.001) and “leaf” alone (*t *= 9.38; *df* = 9; *P *< 0.001). In addition, “fruit” alone attracted significantly more females than the blank control (*t *= 6.51; *df* = 9; *P *< 0.001).

Male responses followed a similar pattern (Fig. 2B). “Fruit + leaf” was preferred over “fruit” alone (*t *= 2.19; *df* = 9; *P *= 0.042), and both “fruit + leaf” (*t *= 8.76; *df* = 9; *P *< 0.001) and “leaf” (*t *= 5.58; *df* = 9; *P *< 0.001) alone were significantly more attractive than the blank control. As with females, “fruit” alone also elicited a stronger response than the blank (*t *= 4.42; *df* = 9; *P *< 0.001).

### Oviposition Experiments with Fruit and Leaf Material

**Fig. 3.**
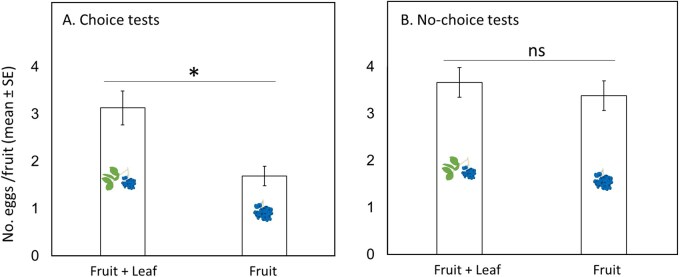
Mean (± SE) number of *Drosophila suzukii* eggs laid per fruit in A) choice and B) no-choice assays comparing fruit + leaf versus fruit alone in semifield (cage) experiments. Ten *D. suzukii* females were released into each cage (Fig. 1B). Significant differences between treatments (*t*-tests) are indicated as follows: *P < 0.05, ns, not significant. *n *= 10.

In semifield (cage) experiments, *D. suzukii* females laid approximately twice as many eggs in fruit clusters with attached leaves than in fruit clusters alone under choice conditions (*t *= 2.51; *df* = 9; *P *= 0.033; Fig. 3A). Under no-choice conditions, however, egg laying did not differ between the 2 treatments (*t *= 0.23; *df* = 18; *P *= 0.818; Fig. 3B). These results indicate that, when given a choice, *D. suzukii* females prefer fruit clusters with leaves for oviposition—a scenario more representative of fruit-ripening conditions, whereas in no-choice situations they show no clear preference.

### Volatile Analyses

**Fig. 4.**
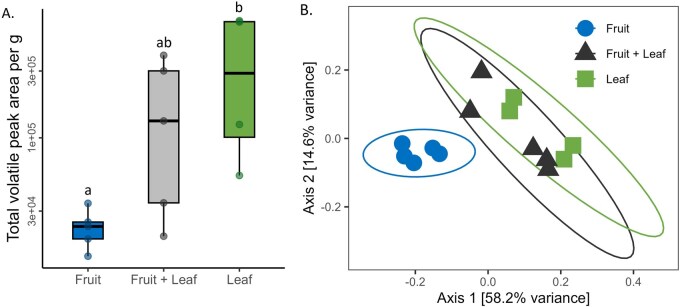
Volatile emission from samples. A) Total volatile emission per gram of fresh weight with letters summarizing differences between sample groups according to Tukey honest significance test. B) Principal coordinate analysis (PCoA) based on Bray–Curtis dissimilarities of volatile blends emitted by blueberry fruit alone, leaves alone, and fruit + leaf combinations. Each point represents a sample. Percentages indicate the variation explained by each axis. *n *= 4 to 5.

We detected 52, 63, and 66 VOCs in the headspaces of the “fruit,” “leaf,” and “fruit + leaf” samples, respectively (Supplementary Table S1). Total volatile emission differed between sample types (*F *= 5.94; *df* = 2, 11; *P *= 0.018). “Leaf” samples emitted 16.6-fold greater total volatile abundance than “fruit” samples (*P *= 0.016). In contrast, total volatile emissions did not differ significantly between “fruit” and “fruit + leaf” samples (*P *= 0.10) or between “leaf” and “fruit + leaf” samples (*P *= 0.48) (Fig. 4A). VOC blends emitted by the samples differed significantly (PERMANOVA: *F *= 6.98; *df* = 2, 11; *R*^2^ = 0.56; *P *= 0.001). Variance was equal between sample types (*P *≥ 0.9). Unsupervised ordination shows 2 distinct clusters separating the “fruit” samples from the “leaf” and “fruit + leaf” samples across the first axis, indicating that the “fruit + leaf” blend is largely dominated by leaf-derived odors (Fig. 4B). The first 2 axes of the PCoA together explained ∼73% of the total variation (Fig. 4B).

Three VOCs were detected exclusively in samples containing fruit: ethyl isovalerate, isobutyl isovalerate, and 3-methylcyclopentyl acetate (Supplementary Table S1). In contrast, samples containing leaves uniquely emitted 15 compounds: (*Z*)-β-ocimene, (*E*)-β-ocimene, (*Z*)-4,8-dimethylnona-1,3,7-triene, (*E*)-4,8-dimethylnona-1,3,7-triene, hexa-hydro-farnesol, 3-ethyl-5-(2-ethylbutyl)-octadecane, myroxide, β-bourbonene, (*E*)-(-)-alpha-bergamotene, citral, (*E*, *E*)-α-farnesene, 2-isohexyl-6-methyl-1-heptene, (-)-β-curcumene, and an unknown compound (Supplementary Table S1). Clustering of volatiles from “fruit,” “leaf,” and “fruit + leaf” samples was also observed in the volatile loading plot (Supplementary Fig. S1), with volatiles dominating in fruit samples located on the left side of the plot and those associated with leaves and the mixed samples on the right side. Among all VOCs detected, 25 were emitted in significantly greater amounts from the “fruit + leaf” treatment compared to “fruit” alone (*P *≤ 0.033; Supplementary Table S1). Of these, 9 compounds (Table 1) were selected for physiological (electroantennography) and behavioral assays, as commercially available standards enabled confirmation of their chemical identities.

### Olfactory Experiments with Synthetic VOCs

**Fig. 5.**
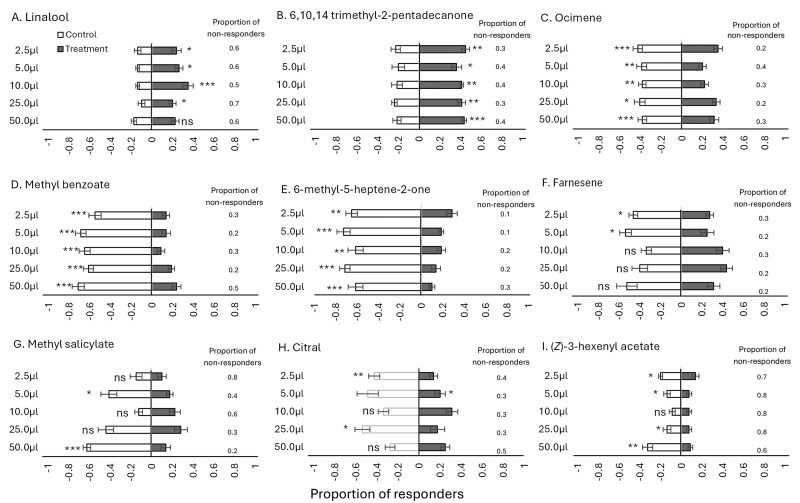
Mean (± SE) proportion of *Drosophila suzukii* females responding to different doses of A) linalool, B) 6,10,14-trimethyl-2-pentadecanone, C) ocimene, D) methyl benzoate, E) 6-methyl-5-hepten-2-one, F) farnesene, G) methyl salicylate, H) citral, and I) (Z)-3-hexenyl acetate in greenhouse experiments. Ten *D. suzukii* females were released into the center of each arena (Fig. 1C). Significant differences between each VOC dose treatment and the blank control (*t*-tests) are indicated as follows: ***P < 0.001, **P < 0.01, *P < 0.05, ns, not significant. *n *= 8.

Of the 9 compounds tested, only 2 were attractive to female *D. suzukii*: linalool and 6,10,14-trimethyl-2-pentadecanone (Fig. 5). For linalool, more females chose the treatment at all doses (2.5 µl, 5 µl, 10 µl, and 25 µl) except the highest at 50 µl (Table 2; Fig. 5A). For 6,10,14-trimethyl-2-pentadecanone, all doses attracted more females than the control (Table 2; Fig. 5B). In contrast, 3 compounds elicited significant repellency: ocimene, methyl benzoate, and 6-methyl-5-hepten-2-one, with more females consistently found in the control across all doses (Table 2; Fig. 5C–E). The remaining 4 VOCs exhibited weaker or inconsistent repellency. Farnesene repelled females at 2.5 and 5 µl, methyl salicylate at 5 and 50 µl, citral at 2.5, 5, and 25 µl, and (*Z*)-3-hexenyl acetate at all doses except 10.0 µl (Table 2; Fig. 5F–I).

**Table 2.** Statistical results for the “Olfactory experiments with synthetic VOCs” trial set presented in Fig. 5

Volatile organic compound (VOC)	Dose (µl)	*t * ^a^	*P*
**Linalool**	2.5	2	0.04
	5	3.42	0.01
	10	4.82	<0.001
	25	3.06	0.02
	50	1.18	0.28
**6,10,14-trimethyl-2-pentadecanone**	2.5	4.47	0.003
	5	2.89	0.02
	10	4.82	0.002
	25	4.65	0.002
	50	9.1	<0.001
**Ocimene**	2.5	6.52	<0.001
	5	5	0.002
	10	4.5	0.003
	25	2.71	0.03
	50	6.07	<0.001
**Methyl benzoate**	2.5	5.95	<0.001
	5	6.68	<0.001
	10	10.56	<0.001
	25	6.86	<0.001
	50	4.86	<0.001
**6-methyl-5-hepten-2-one**	2.5	3.77	0.007
	5	6.91	<0.001
	10	4.27	0.004
	25	8.21	<0.001
	50	6.69	<0.001
**Farnesene**	2.5	2.5	0.03
	5	3.37	0.01
	10	−0.78	0.46
	25	−0.36	0.73
	50	1.46	0.19
**Methyl salicylate**	2.5	0.73	0.49
	5	2.37	0.05
	10	−1.87	0.10
	25	1.63	0.15
	50	7.04	<0.001
**Citral**	2.5	4.95	0.002
	5	2.31	0.05
	10	0.31	0.76
	25	2.8	0.03
	50	0.36	0.73
**(Z)-3-hexenyl acetate**	2.5	2.38	0.05
	5	2.38	0.05
	10	0.36	0.73
	25	2.38	0.05
	50	4.77	0.002

a Degrees of freedom (*df*   ) = 7 for all tests.

**Fig. 6.**
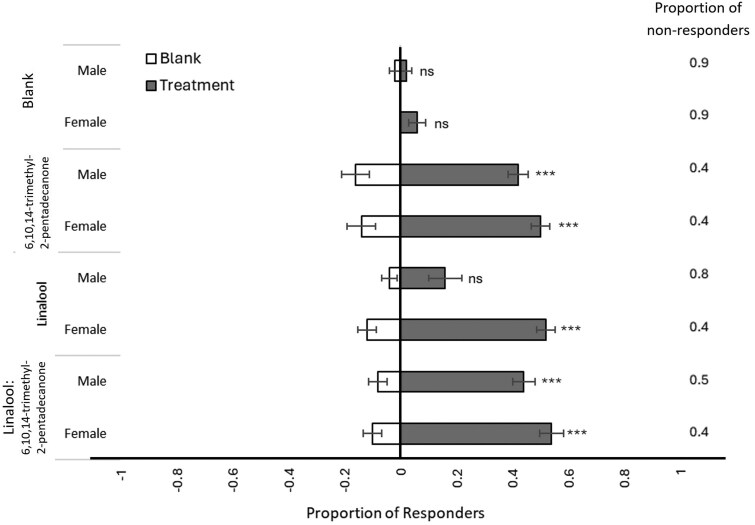
Mean (± SE) proportion of *Drosophila suzukii* females and males responding to linalool, 6,10,14-trimethyl-2-pentadecanone, and their mixture. Ten *D. suzukii* adults (5 males and 5 females) were released into the center of each arena (Fig. 1A). Significant differences between each VOC treatment and the blank control (*t*-tests) are indicated as follows: ***P < 0.001, ns, not significant. *n *= 10.

When tested individually and as a blend, both attractive VOCs significantly increased female captures compared to the control: linalool (*t *= 7.75; *df* = 9; *P *< 0.001), 6,10,14-trimethyl-2-pentadecanone (*t *= 5.01; *df* = 9; *P *< 0.001), and the blend (*t *= 7.57; *df* = 9; *P *< 0.001) (Fig. 6). Male responses were also significant for 6,10,14-trimethyl-2-pentadecanone (*t *= 3.28; *df* = 9; *P *= 0.001) and the blend (*t *= 5.51; *df* = 9; *P *= 0.001) (Fig. 6). By contrast, the blank elicited very low and nonsignificant behavioral responses (Fig. 6).

### Electroantennographic Assays

**Fig. 7.**
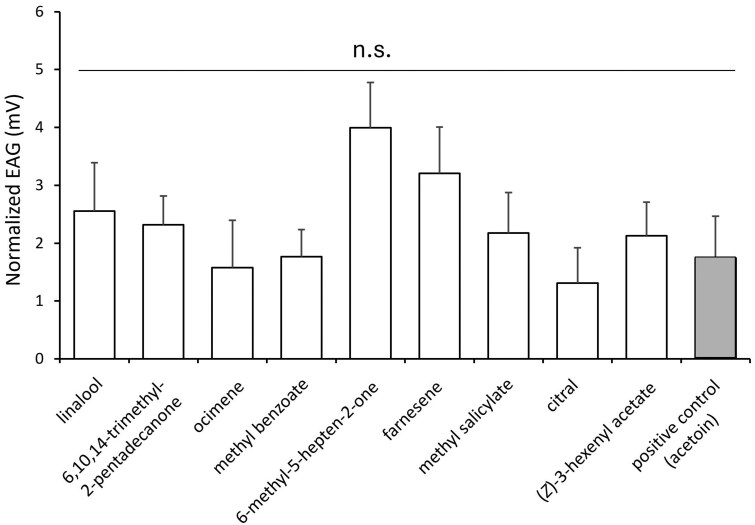
Mean (± SE) electroantennogram (EAG) responses of female *Drosophila suzukii* to selected volatile compounds shown in Table 1. Values were normalized by subtracting the responses to the air (negative) controls. There were no significant differences; ns, not significant. *n *= 10.

All tested VOCs elicited EAG responses that did not differ significantly from those to acetoin, the positive control (*F *= 1.35; *df* = 9, 91; *P *= 0.221) (Fig. 7).

## Discussion

Frugivorous insects, including *D. suzukii*, likely use a hierarchical sensory strategy to locate hosts, relying first on vegetative plant volatiles to identify suitable habitats from a distance and then shifting to more specific fruit- or microbe-derived cues for precise host location (Quilici et al. 2014). Leaf odors may serve as long-range cues for *D. suzukii* because the greater biomass and surface area of foliage produce larger, more easily detectable odor plumes than the smaller, discrete sources emitted by individual fruit (Webster and Cardé 2017). Consequently, *D. suzukii* likely depends on this combination of broad habitat cues (leaves) and specific host cues (fruit and microbes) to navigate effectively to oviposition sites (Cloonan et al. 2018). The present study provides laboratory and semifield evidence that *D. suzukii* is attracted to blueberry leaf volatiles and that the presence of leaves enhances both attraction to and oviposition on blueberry fruit. Leaves were more fragrant than fruit on a per gram basis, perhaps a consequence of the higher surface area of leaves relative to fruit. Complementary behavioral and EAG assays revealed that 2 VOCs (linalool and 6,10,14-trimethyl-2-pentadecanone) were attractive to *D. suzukii* and elicited antennal responses. These VOCs were detected in higher quantities in fruit-plus-leaf blends than in fruit-alone blends.

Our study demonstrates that VOCs from host leaves can act in combination with fruit-emitted VOCs to increase *D. suzukii* attraction and oviposition. These findings are consistent with previous work showing that *D. suzukii* can detect and are attracted to VOCs emitted by host-plant leaves, such as strawberry (Keesey et al. 2015), and that leaf odors can synergize with other host-associated volatile cues to increase attraction (Keesey et al. 2015, Bolton et al. 2019, 2021, Piñero et al. 2019). For example, the strawberry leaf odor β-cyclocitral synergizes with the ripening-fruit odors ethyl acetate and ethyl hexanoate (Bolton et al. 2019), as well as with the microbial odor isoamyl acetate (Piñero et al. 2019), to enhance female attraction compared to nonleaf odors alone.

Although β-cyclocitral was not detected in the headspace of blueberry leaves in the present study, 2 VOCs collected from blueberry leaf headspace (linalool and 6,10,14-trimethyl-2-pentadecanone) elicited attraction and EAG responses in *D. suzukii* females, and their combination further enhanced attraction. Of the 2, linalool is a widespread monoterpene alcohol that plays key roles in plant–insect interactions (He et al. 2019, Zhang et al. 2023). Its emission from leaves can occur constitutively at low levels but is often induced by biotic or abiotic stresses, including herbivory (Rodriguez-Saona et al. 2009, Spinelli et al. 2011). In blueberries, we found that linalool is emitted in greater quantities from leaves than from fruits, suggesting that this compound may function as a long-range cue for *D. suzukii* during habitat selection due to its large production quantity. Previous studies have documented antennal responses of *D. suzukii* to linalool (Abraham et al. 2015, Keesey et al. 2015, Revadi et al. 2015, Kirkpatrick et al. 2018), and behavioral assays have demonstrated that this compound can be attractive to the fly (Dewitte et al. 2021, Baena et al. 2022).

Compared to linalool, the ketone 6,10,14-trimethyl-2-pentadecanone (also known as hexahydrofarnesyl acetone) is a less ubiquitous plant VOC, and its role in plant–insect interactions remains less understood. This compound has been detected in the headspace of rice (Ghaninia and Amooghli Tabari 2016), corn (Konstantopoulou et al. 2004), tomato (Ibrahim et al. 2018), and winter wheat (Strand et al. 2025). Interestingly, *Bracon cephi* (Gahan) and *Bracon lissogaster* Muesebeck, 2 parasitoids of the wheat stem sawfly (*Cephus cinctus* Norton), exhibited dose-dependent behavioral and electrophysiological responses to this compound (Strand et al. 2025), indicating it may serve as an important plant-derived cue across diverse insect taxa. Together with β-cyclocitral and linalool, these findings suggest that 6,10,14-trimethyl-2-pentadecanone may act as an attractive leaf odor for *D. suzukii*, warranting further evaluation under field conditions.

Leaf VOCs such as β-cyclocitral, linalool, and 6,10,14-trimethyl-2-pentadecanone may serve as important long-range cues guiding *D. suzukii* to ripening host fruits still attached to plants. Fruit and microbial VOCs likely then facilitate short-range attraction and provide oviposition cues (Cloonan et al. 2018). Further work is needed to test the hypothesis that leaf VOCs primarily act as long-range attractants during habitat selection, whereas fruit and microbial VOCs function as short-range cues during host location. The observed preference for fruit associated with leaf material supports the ecological adaptation that distinguishes *D. suzukii* from *Drosophila melanogaster* Meigen, which primarily exploits fallen, overripe, and fermenting fruit.

The role of leaf VOCs in this adaptation is further supported by Keesey et al. (2015), who showed that the “ab3” olfactory sensory neuron (OSN) in *D. suzukii* is sensitive to β-cyclocitral but responds weakly to the fermentation odors methyl and ethyl hexanoate. In contrast, the ab3 OSN in *D. melanogaster* responds strongly to methyl and ethyl hexanoate but weakly to β-cyclocitral. This suggests that the odorant receptor OR22a housed within the ab3 OSN is tuned to leaf odors in *D. suzukii* but to fermentation odors in *D. melanogaster*. Moreover, Duménil et al. (2025b) found differences in antennal lobe structure between the 2 species: the DM4 and DL5 glomeruli, which are innervated by OSNs tuned to ripening-fruit odors and green leaf volatiles, respectively (Hallem and Carlson 2006, Keesey, 2022), are larger in *D. suzukii*. Given that linalool and 6,10,14-trimethyl-2-pentadecanone are newly identified as attractive leaf VOCs, their detection on female antennae and processing in the antennal lobe merit further investigation.

The blend of blueberry fruit and leaf volatiles was complex, and we tested 9 selected compounds that were emitted in higher quantities from this blend compared to the fruit-only blend. Among these, ocimene, methyl benzoate, and 6-methyl-5-hepten-2-one were repellent to *D. suzukii*. Although methyl benzoate is a known repellent of *D. suzukii* (Feng and Zhang 2017, Gale et al. 2024), the behavioral effects of ocimene and 6-methyl-5-hepten-2-one are less well known. It is likely that *D. suzukii* responds to a blend of attractive and aversive VOCs during habitat selection and host location, and that the relative amounts and ratios of these VOCs ultimately shape behavioral responses. For example, a blend of volatiles from *Vicia faba* L. containing compounds identified as repellents in dose–response bioassays was attractive to the black bean aphid (*Aphis fabae* Scopoli) (Webster et al. 2010). We acknowledge that we tested only 9 of the 66 VOCs identified in the fruit and leaf blend because dose–response experiments are time-consuming. Using methods such as coupled gas chromatography-electroantennographic detection could help identify additional behaviorally active VOCs.

Other factors may also have influenced attraction and oviposition in our assays. For example, undetected microbial VOCs could have contributed to the observed responses. Although isoamyl acetate and isobutyl acetate, 2 microbial odors associated with *D. suzukii* (Scheidler et al. 2015), were not identified from blueberry leaf or fruit headspace in this study, they have been reported from mature blueberry fruit (Urbaneja-Bernat et al. 2021). Microbial activity on leaves may have affected VOC emissions, and leaf presence could have altered the microenvironment around fruit, influencing volatile emission rates or humidity. Leaf color could have further contributed to attraction, as visual cues can synergize with odors to increase *D. suzukii* attraction (Bolton et al. 2021). For example, β-cyclocitral synergized with yellow color, while fruit odors synergized with red, black, yellow, green, blue, and purple (Bolton et al. 2021). Although we did not explicitly test color–odor interactions, increased visual attraction in leaf treatments could partly explain the enhanced behavioral responses. Physiological state may also play a role, as factors such as age, mating status, or feeding status can modulate olfactory-driven behaviors in insects (Grunwald Kadow 2019).

In conclusion, our study demonstrates that blueberry leaves significantly enhance *D. suzukii* attraction to and oviposition on blueberry fruit. This effect likely results from interactions between fruit odors and other factors, including leaf volatiles such as linalool and 6,10,14-trimethyl-2-pentadecanone, microbial VOCs, and color–odor associations. Future research should investigate how linalool and 6,10,14‑trimethyl‑2‑pentadecanone interact with other host-associated odors, how these compounds are detected by the antennae, and how they are processed in specific olfactory glomeruli (eg DM4, DL5) associated with leaf and fruit odor detection. We acknowledge that our studies were conducted under controlled conditions at doses that may not reflect field realities, and that field experiments across a range of VOC doses are needed to evaluate their performance relative to existing lures. Nonetheless, these findings advance our understanding of the chemical ecology of *D. suzukii* and may inform the development of more selective attractants for improved management of this economically important pest.

## Supplementary Material

ieag053_Supplementary_Data
