# Supplementary material for: Role of leaf volatiles in spotted-wing drosophila (Diptera: Drosophilidae) attraction to blueberry fruits
Source: J Insect Sci. 2026 Jun 16;26(3):ieag053. doi: 10.1093/jisesa/ieag053 (PMC13271246; doi:10.1093/jisesa/ieag053)
Supplement: ieag053_Supplementary_Data [file ieag053_supplementary_data.zip › Fig S1.docx]

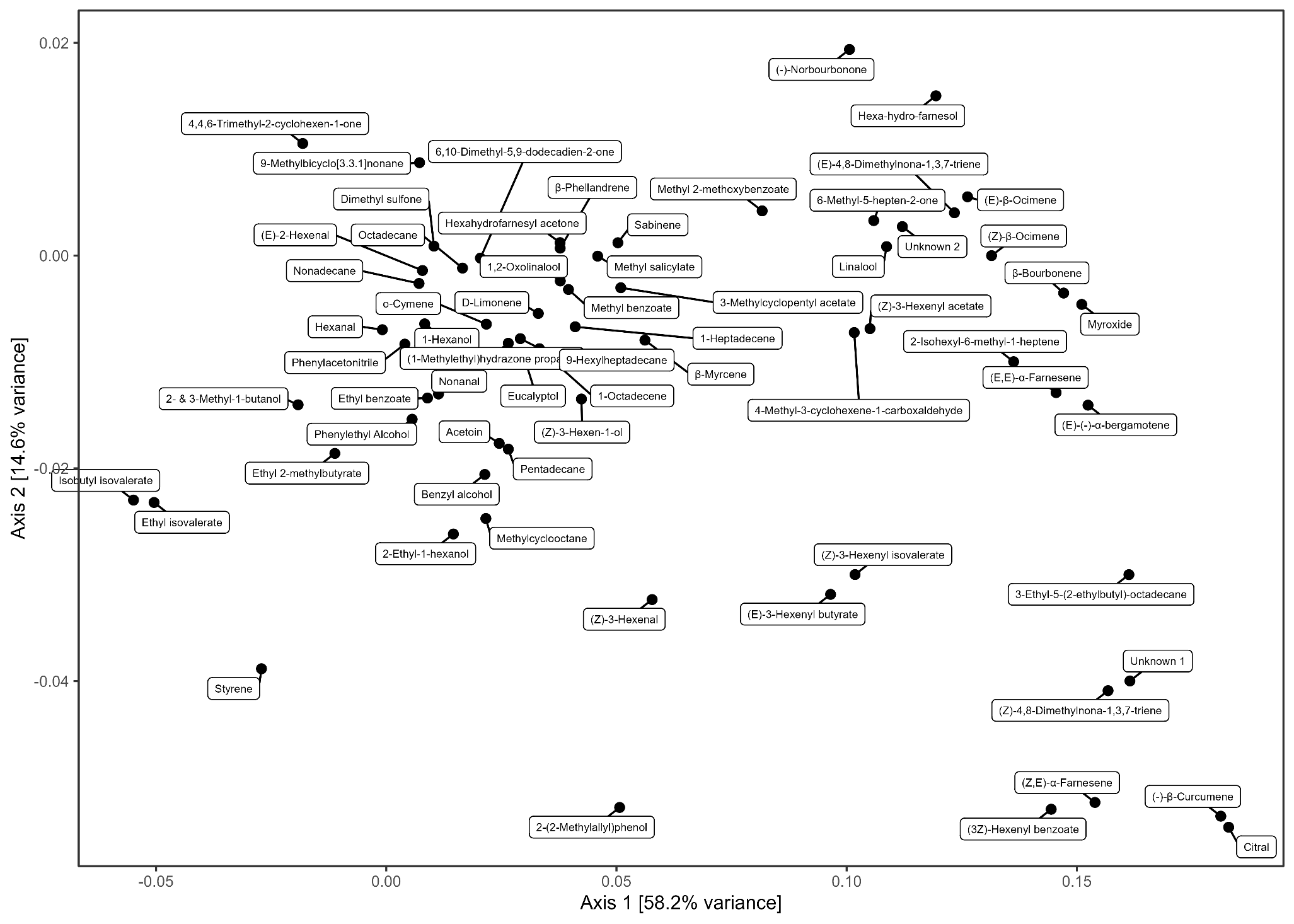


Fig S1. Volatile loading plot of the principal coordinate analysis (PCoA) of blueberry fruit, leaves, and fruit and leaves samples. Volatiles are represented as points with labelled identities (see Table S1 for identity confidence). Volatiles in closer proximity to one another share abundance patterns in samples. Farther distance from the PC axis origin indicates the volatile has a greater influence on the PC axis.
